# Factors contributing to non-compliance with on-demand treatment guidelines in hereditary angioedema

**DOI:** 10.1186/s13223-025-00969-0

**Published:** 2025-05-21

**Authors:** Stephen D. Betschel, Jonny Peter, William Lumry, Hilary Longhurst, Constance H. Katelaris, Sally van Kooten, Markus Heckmann, Neil Malloy, Julie Ulloa, Sherry Danese, Markus Magerl

**Affiliations:** 1 Department Division Director Clinical Immunology and Allergy, Staff Clinical Immunologist and Allergist, University of Toronto, St. Michael’s Hospital, 36 Toronto St, Suite 700, Toronto, ON M5C 2C5 Canada; 2 University of Cape Town, Cape Town, South Africa; 3 AARA Research Center, Dallas, TX USA; 4 Department of Immunology, University of Auckland, Auckland City Hospital, Te Toka Tumai, Auckland, New Zealand; 5 Campbelltown Hospital and Western Sydney University, Sydney, NSW Australia; 6 KalVista Pharmaceuticals, Inc, Cambridge, MA USA; 7 Summit Global Health, Old Lyme, CT USA; 8 Outcomes Insights, Agoura Hills, CA USA; 9 Institute of Allergology, Charité—Universitätsmedizin Berlin, Berlin, Germany; 10 Fraunhofer Institute for Translational Medicine and Pharmacology ITMP, Immunology and Allergology, Berlin, Germany

**Keywords:** Hereditary angioedema, On-demand treatment, Guideline recommendations, Compliance, Survey

## Abstract

**Background:**

Hereditary angioedema (HAE) is a rare genetic disorder characterized by painful and potentially life-threatening tissue swelling due to a deficiency or dysfunction of the C1 esterase inhibitor protein. Despite the availability of comprehensive on-demand treatment guidelines, compliance to guideline recommendations remains suboptimal, resulting in persisting unmet need.

**Methods:**

This observational, online survey was conducted between September 6, 2022, and October 19, 2022 to understand the behaviors and perspectives of individuals in the US with hereditary angioedema (HAE). Participants were recruited by the US Hereditary Angioedema Association and were eligible if they were US residents with clinician-diagnosed HAE type I or II and had experienced at least one HAE attack. The survey included multiple-choice, rank-order, and scale-based responses using a 5-point Likert scale for agreement and an 11-point Likert scale for anxiety. Statistical analysis was performed using Microsoft Excel, summarizing continuous variables as means, medians, and ranges, and categorical variables as frequency distributions and percentages.

**Results:**

A total of 107 out of 155 participants completed the survey (mean age = 41 years; 80.4% female). Half of the respondents used both prophylaxis and on-demand therapy, while the other half used on-demand therapy only. Icatibant was the most commonly used on-demand treatment (78.5%). The survey revealed that 57% of respondents did not treat all HAE attacks, and only 14% treated attacks immediately. Delays in treatment were common, with a mean time to treatment of 2.4 h, and younger patients were less likely to carry on-demand treatment. Reasons for delaying treatment included the perceived severity of the attack, lack of on-demand treatment availability, and pain associated with treatment. Additionally, 32.7% of respondents experienced the return of an HAE attack after initial treatment, with those delaying treatment more likely to experience recurrence. The survey also found that delayed treatment led to more severe attacks and longer recovery times, impacting work, social activities, and overall quality of life.

**Conclusions:**

Although guidelines recommend early treatment of HAE attacks, many respondents do not treat immediately. This finding underscores the importance of incorporating open patient-physician communication to improve guideline compliance and the management of HAE.

**Supplementary Information:**

The online version contains supplementary material available at 10.1186/s13223-025-00969-0.

## Introduction

Hereditary angioedema (HAE) is a rare genetic disorder caused by a deficiency or dysfunction of the C1 esterase inhibitor (C1-INH) protein, leading to the uncontrolled activation of the kallikrein-kinin system [1]. HAE is characterized by unpredictable, painful, and debilitating attacks of tissue swelling in various locations of the body that can be life-threatening depending on the location affected [2]. Current management of HAE involves the use of parenteral medications for on-demand treatment of angioedema events (HAE attacks) with the objective of relieving symptoms as quickly and completely as possible. To help reduce the frequency and potentially the severity of HAE attacks, it is also recommended that appropriate patients receive long-term prophylaxis [3, 4].

The World Allergy Organization/European Academy of Allergy and Clinical Immunology 2021 updated HAE guidelines recommend that patients consider the treatment of all attacks, regardless of location or severity [3], treat attacks as early as possible to minimize their duration and severity, and always carry enough on-demand medication to treat at least two attacks. A summary of key recommendations from current international, US and Canadian HAE guidelines for on-demand treatment is presented in Table 1. The recommendations for on-demand therapy are consistent across the three guidelines.

**Table 1:** Summary of key recommendations for on-demand treatment

Guideline
World Allergy Organization/European Academy of Allergy and Clinical Immunology (2021) [3]
• We recommend that all attacks are considered for on-demand treatment • We recommend that any attack affecting or potentially affecting the upper airway is treated • We recommend that attacks are treated as early as possible • We recommend that attacks are treated with either IV C1 inhibitor, ecallantide or icatibant • We recommend that all patients have sufficient medication for on-demand treatment of at least two attacks and carry on-demand medication at all times
US Hereditary Angioedema Association Medical Advisory Board (2020) [2]
• Patients must have ready access to effective on-demand medication to administer at the onset of an HAE attack. An FDA-approved on-demand HAE medication (ecallantide, icatibant, pdC1-INH, or rhC1-INH) should be used as first-line treatment for attacks whenever possible • On-demand treatment of HAE attacks should be self-administered (or administered by a caregiver) whenever feasible except when treating with ecallantide that needs to be administered by a health care provider • All HAE attacks are eligible for treatment irrespective of the location of the swelling or the severity of the attack
International/Canadian Hereditary Angioedema Guideline (2019) [5]
• Effective therapy should be used for the acute treatment of attacks of angioedema to reduce duration and severity of attacks • IV pdC1-INH is an effective therapy for the acute treatment of attacks • Icatibant is an effective therapy for the acute treatment of attacks • Ecallantide is an effective therapy for the acute treatment of attacks • IV rhC1-INH is an effective therapy for the acute treatment of attacks • Attenuated androgens should not be used for the acute treatment of attacks • Tranexamic acid should not be used for the acute treatment of attacks • Frozen plasma could be used for acute treatment of attacks if other recommended therapies are not available • Attacks should be treated early to reduce morbidity and mortality • All attacks of angioedema involving the upper airway are medical emergencies and must be treated immediately

FDA, US Food and Drug Administration; HAE, hereditary angioedema; IV, intravenous; pdC1-INH, plasma-derived C1 esterase inhibitor; rhC1-INH, recombinant human C1 esterase inhibitor

Despite the availability of comprehensive on-demand treatment guidelines, recent real-world studies have demonstrated that implementation appears to have been ineffective, resulting in persisting unmet need [6–9].

In order to gain further insight into the unmet needs of patients receiving HAE treatment, we conducted a survey to explore the management patterns of individuals living with HAE. The main objective of this survey was to analyze patient behaviors and attitudes regarding on-demand treatment for managing HAE attacks, and the extent to which their treatment aligns with established guidelines. Our analysis focuses on patients’ awareness of the impact of early treatment, treatment delays, or non-treatment of HAE attacks; compliance with early treatment of attacks; and attack recurrence.

## Methods

### Survey development

We conducted an observational, online survey to characterize the behaviors and perspectives of people in the US with HAE. The survey questions were original and included multiple-choice questions, rank-order questions (order of importance 1–5), and scale-based responses using a symmetrical 5-point Likert scale of agreement with presented statements (from “strongly disagree” to “strongly agree”), consistent with established self-reporting survey methodology [10], and an 11-point Likert scale to evaluate anxiety (0: not anxious; 10: extremely anxious).

### Participants

Patients completed the online survey between September 6, 2022 and October 19, 2022. Participants were recruited by the US Hereditary Angioedema Association and were eligible if they were US residents with self-reported, clinician-diagnosed HAE type I or II, and had experienced 1 or more HAE attacks in their lifetime. There were no exclusionary parameters; however, the survey was designed to include an even distribution of respondents based on HAE therapy use (on-demand therapy only or a combination of long-term prophylaxis and on-demand therapy). Prior to survey initiation, all materials were reviewed, and a waiver was granted by Advarra, an independent review board. All participants provided informed consent upon entry to the online survey for their data to be used anonymously or in aggregate prior to entering the survey. Participants completed the survey online via a secure web portal/electronic data capture system that took approximately 20 min to complete.

### Statistical analysis

Statistical analysis was performed using Microsoft Excel (Microsoft, Redmond, WA, USA). The analysis plan specified continuous variables to be summarized as means, medians, and ranges, and categorical variables as frequency distributions and percentages.

## Results

### Characteristics of respondents

A total of 155 individuals with HAE initiated the online survey, with 107 completing the survey (“respondents”) and 48 non-completers, resulting in a completion rate of 69%. Respondent characteristics have been previously reported [11] and are presented in Table 2.

**Table 2:** Respondent characteristics

Characteristics	All Respondents *N* = 107
**Mean age**,** y (SD)**	41 (14.6)
**Age categories**,** n (%)**	
< 18 18–24 25–34 35–44 45–54 55–64 65–74 75+	2 (1.9%) 12 (11.2%) 29 (27.1%) 22 (20.6%) 21 (19.6%) 15 (14.0%) 4 (3.7%) 2 (1.9%)
**Gender**,** Female**,** n (%)**	86 (80.4%)
**Treatment type**,** n (%)**	
Prophylaxis + On-demand On-demand only	54 (50.5%) 53 (49.5%)
**Current prophylactic treatment**,** n (%)**	
Lanadelumab Berotralstat C1 esterase inhibitor (subcutaneous) Androgens/steroids C1 esterase inhibitor (intravenous) Not taking prophylactic treatment	31 (29.0%) 7 (6.5%) 7 (6.5%) 5 (4.7%) 4 (3.7%) 53 (49.5%)
**Current primary on-demand treatment**,** n (%)**	
Icatibant C1 esterase inhibitor (recombinant) C1 esterase inhibitor (human) Ecallantide	84 (78.5%) 13 (12.1%) 9 (8.4%) 1 (0.9%)

SD, standard deviation

### Compliance with recommendations to treat all HAE attacks

**Fig. 1:**
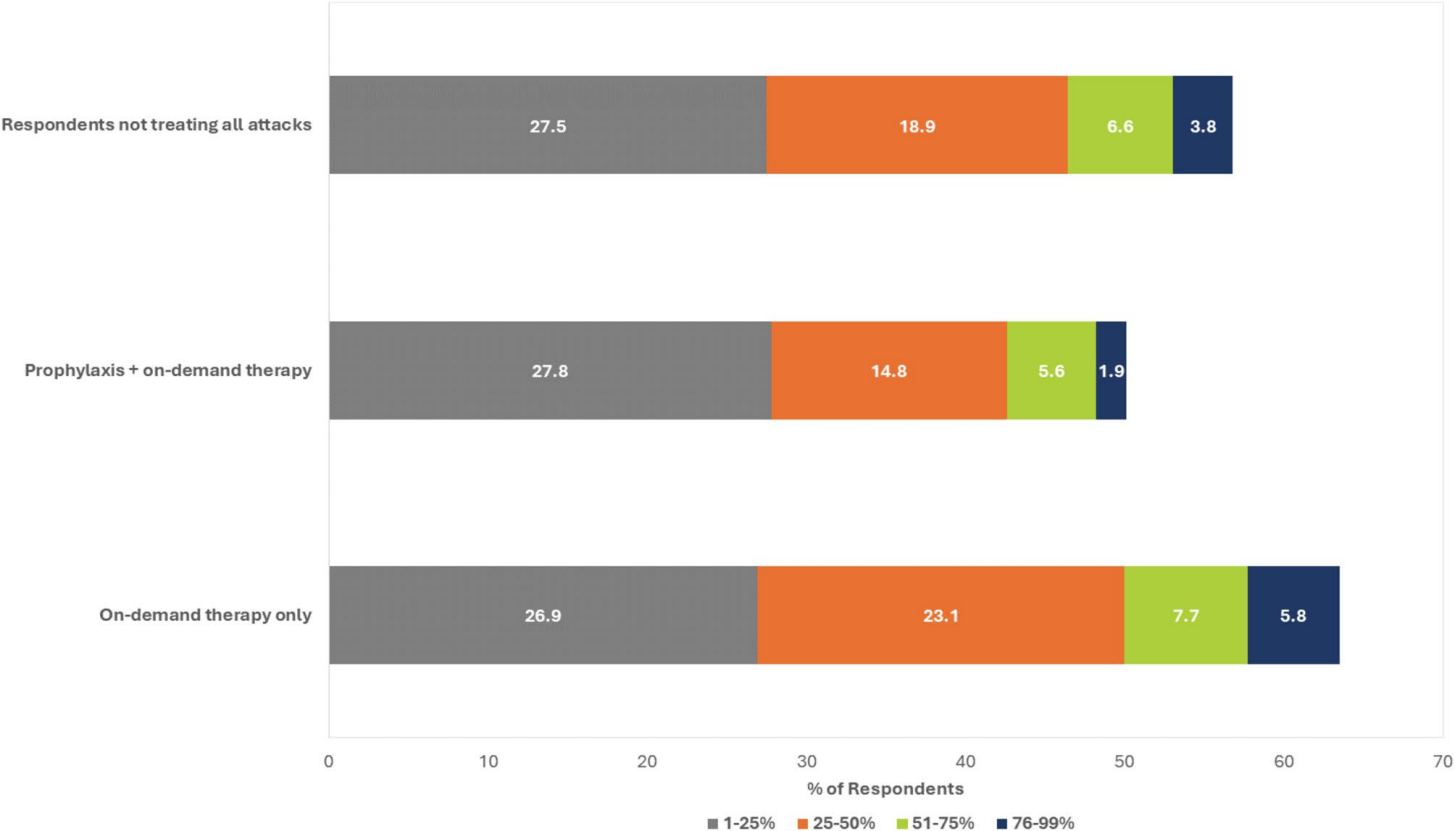
Proportion of HAE attacks that were not treated by respondents

When respondents were asked what percentage of HAE attacks they did not treat, it was previously reported that the majority (57%) did not treat all HAE attacks [12]. The current analysis assessed the proportion of HAE attacks treated by respondents who did not treat all attacks, including those using prophylaxis + on-demand therapy and those using on-demand therapy only (Fig. 1).

### Compliance with recommendations to treat HAE attacks as early as possible

As previously reported, 14% of respondents reported that they immediately treat (within 30 min of recognition of attack onset) all HAE attacks (9.4% of on-demand therapy only patients; 18.5% of those on prophylaxis + on-demand therapy) [12]. Based on the current analysis, this group tended to be older than mean age (44.5 years vs. 40.4 years for those who did not treat immediately) and were more likely to be on long-term prophylaxis (66.8% vs. 47.7% for those who did not treat immediately). This group was also less anxious about using their on-demand treatment (mean [SD]: 2.1 [3.3] vs. 4.6 [3.3] on 0–10 anxiety scale).

Most respondents (86%) reported that they do not always immediately treat HAE attacks, with a mean time to treatment of 2.4 h (median 1 h) for the entire group and 3.7 h (median 2 h) among those aged 24 years or younger. A majority of respondents (57.0%) waited 1 h or longer to initiate on-demand treatment; these patients waited a mean of 3.9 h (median 2 h) to treat. This group was also less likely to carry on-demand treatment when away from home (58.9% vs. 70.5% of patients who treated within [< ] 60 min of an attack) and was more likely to not treat their attacks (27.5% vs. 9.7% of patients who treated within 60 min of an attack).

Respondents ranked the most important reasons for waiting to treat an attack using rankings from 1 (highest) to 5 (lowest) for any given rank. The most important reasons cited by those who waited 1 h or longer to initiate on-demand treatment were the attack not being considered severe enough to treat (86.9%), not having on-demand treatment with them (59%), and their on-demand treatment being too painful (32.8%).

Among those who waited 1 h or longer to initiate on-demand treatment, 59% expressed moderate or extreme anxiety (rating of 4–10 on anxiety scale) when anticipating use of their current on-demand treatment compared with 34.7% of patients who treated within 60 min of an attack).

### Compliance with recommendations to carry on-demand treatment at all times

Respondents were asked if they always carry their HAE on-demand treatment when they are away from home, with 6% carrying none of the time, 22% carrying 1–25% of the time, 15% carrying 26–50% of the time, 4% carrying 51–75% of the time, 17% carrying 76–99% of the time, and 36% carrying 100% of the time). In the prophylaxis + on-demand group, 6% carried their on-demand treatment with them none of the time, 13% carried 1–25% of the time, 22% carried 26–50% of the time, 7% carried 51–75% of the time, 17% carried 76–99% of the time, and 35% carried 100% of the time. In the on-demand therapy only group, 6% carried their on-demand treatment with them none of the time, 32% carried 1–25% of the time, 8% carried 26–50% of the time, 17% carried 76–99% of the time, and 38% carried 100% of the time. Patients younger than 40 years of age reported carrying on-demand treatment with them less often than patients 40 years of age and older (57% vs. 72% of the time). None of the respondents 24 years of age or younger (*n* = 14) reported always carrying their on-demand treatment with them; on average, these respondents carried their on-demand treatment with them 41% of the time.

Patients reported traveling a mean of 3.5 h without their on-demand treatment, with more than 50% traveling longer than 1 h without on-demand treatment and 15% traveling fewer than 6 h without their on-demand treatment, including 21% of the on-demand therapy only group.

**Fig. 2:**
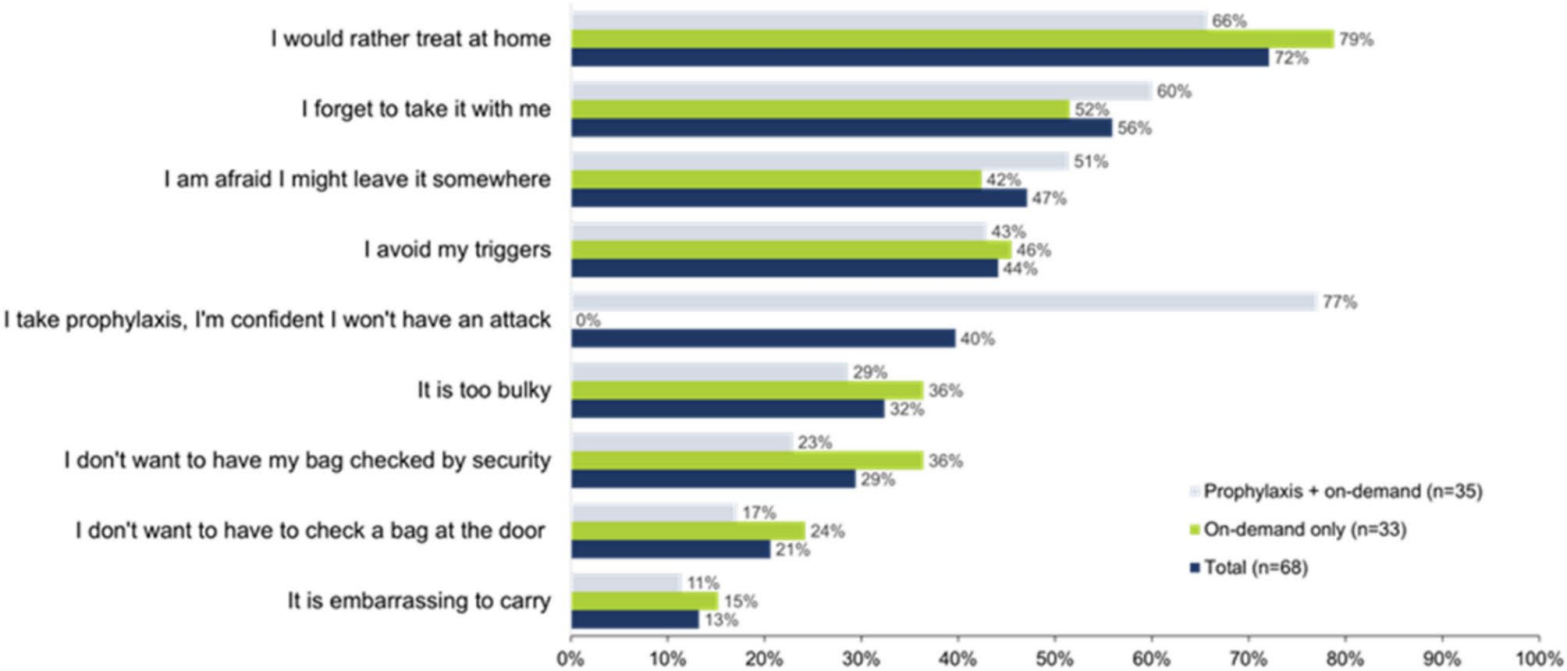
Reasons for not carrying on-demand treatment^a^ ^a^Survey participants who indicated that they do not always carry an HAE on-demand treatment were asked to select from a list of reasons they do not take on-demand treatment when away from home HAE, hereditary angioedema

Respondents who indicated that they do not always carry an HAE on-demand treatment were asked to select from a list of reasons they do not take on-demand treatment when away from home using rankings from 1 (highest) to 5 (lowest) for any given rank. Reasons for not carrying on-demand treatment are presented in Fig. 2; **72**% of all respondents reported that they would rather treat at home while 56% cited forgetfulness as the reason for not carrying on-demand treatment. In addition, 44% reported adjusting their daily lives to avoid triggers, rather than carrying on-demand treatment when away from home. 77% of patients on long-term prophylaxis state that they do not carry their required medication with them at all times. On the one hand, this finding may reflect the high level of confidence in the effectiveness of the currently available therapies for long-term prophylaxis; on the other hand, this behavior is worrying because even the well-tolerated and very efficient long-term prophylaxis therapies cannot guarantee complete absence of breakthrough attacks.

### HAE attack recurrence

**Fig. 3:**
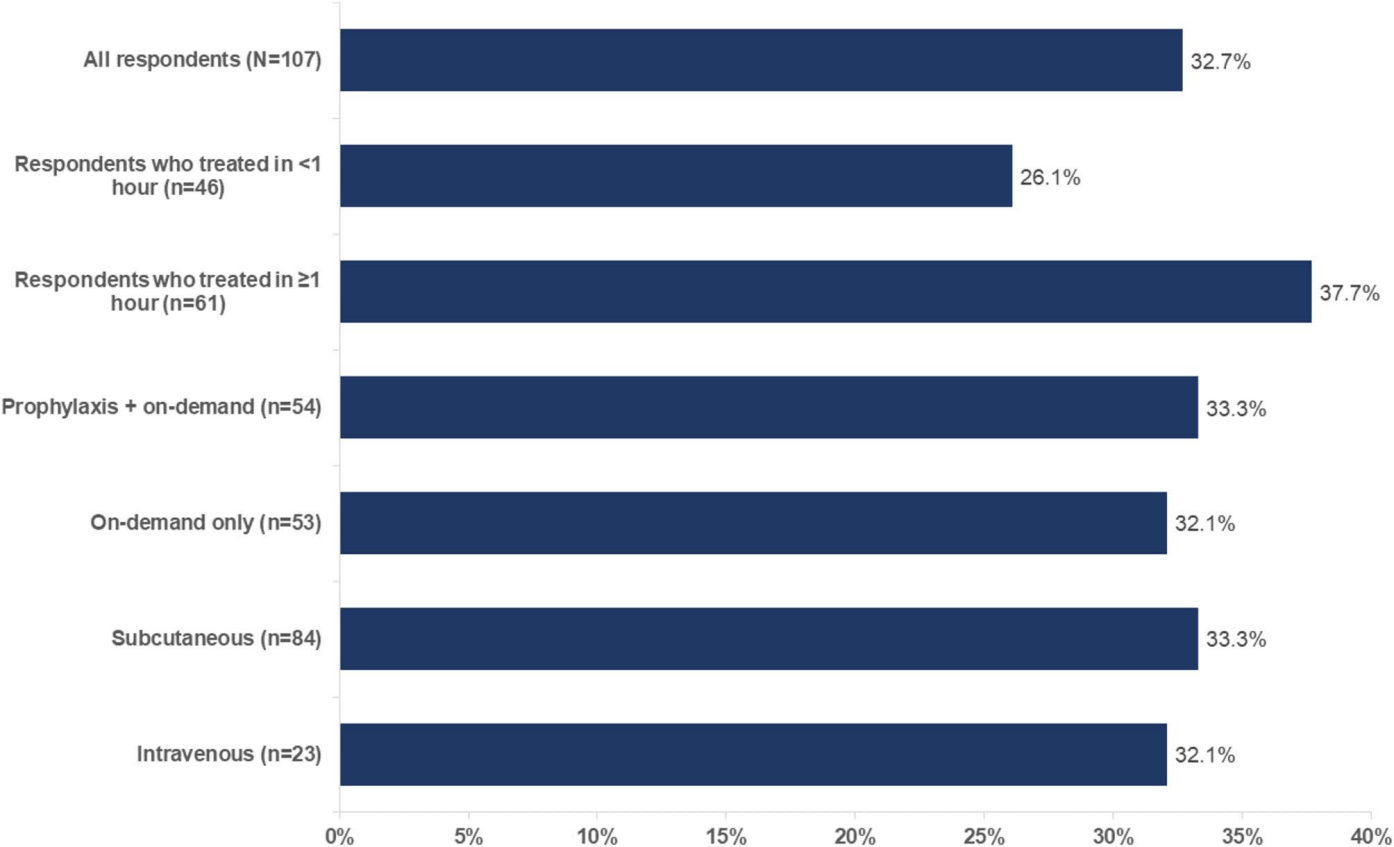
Proportion of patients who experienced return of an HAE attack after initial use of on-demand treatment

Respondents were asked if they experienced the return of an HAE attack after initial use of on-demand treatment; 35 respondents (32.7%) reported experiencing the return of their HAE attack [12] (26.1% and 37.7% of patients experienced return of an HAE attack when HAE attacks were initially treated within 1 h and after ≥ 1 h, respectively; Fig. 3).

**Fig. 4:**
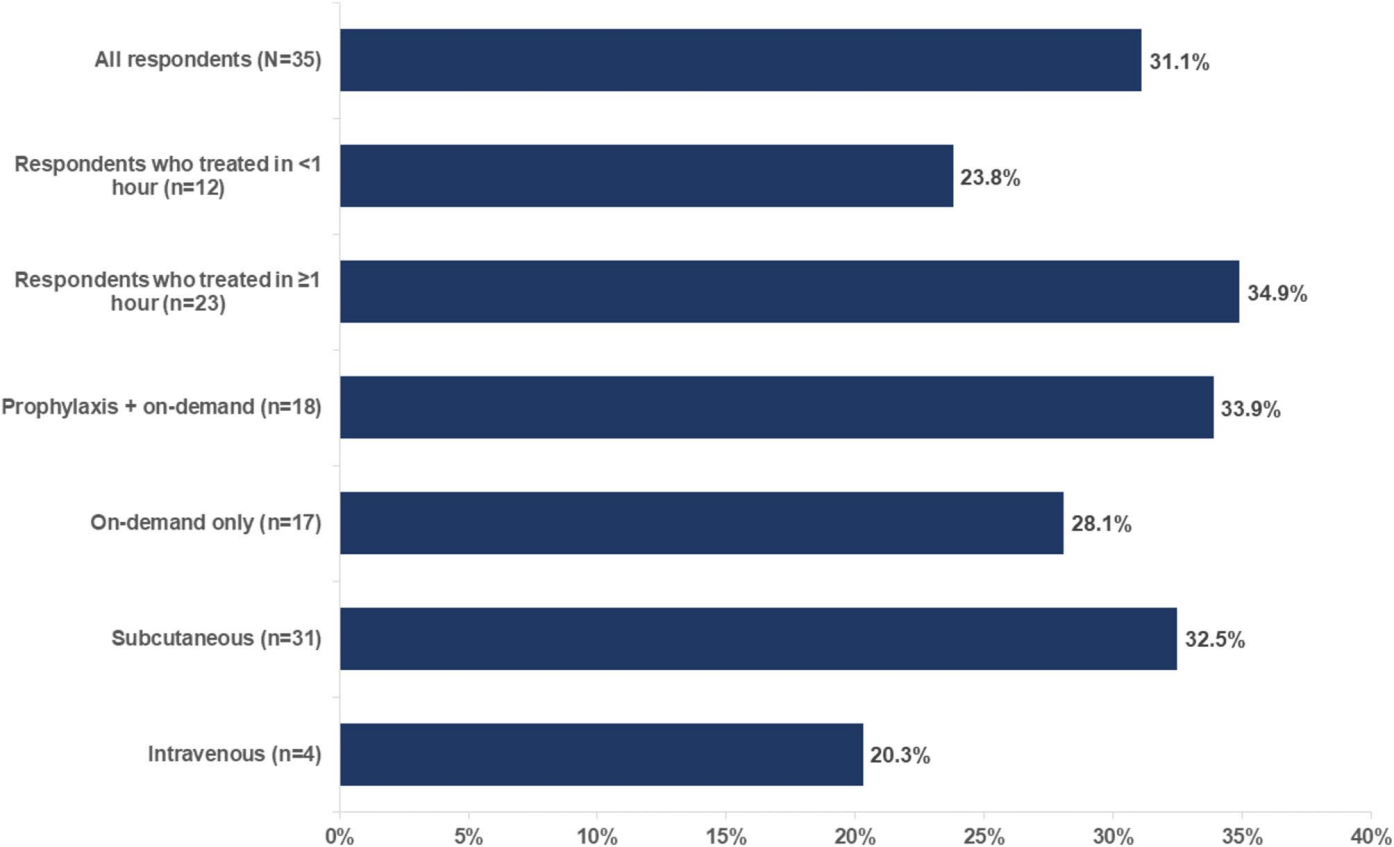
Percentage of time respondents^a^ reported experiencing the return of an HAE attack after taking on-demand treatment ^a^All respondents were those who experienced return of HAE attack after taking on-demand treatment

On average, respondents reported experiencing the return of an HAE attack 31.1% of the time after taking on-demand treatment (Fig. 4). Respondents who waited 1 h or longer to initiate on-demand treatment reported experiencing a return attack 34.9% of the time versus 23.8% for those who treated within 60 min of an attack.

**Fig. 5:**
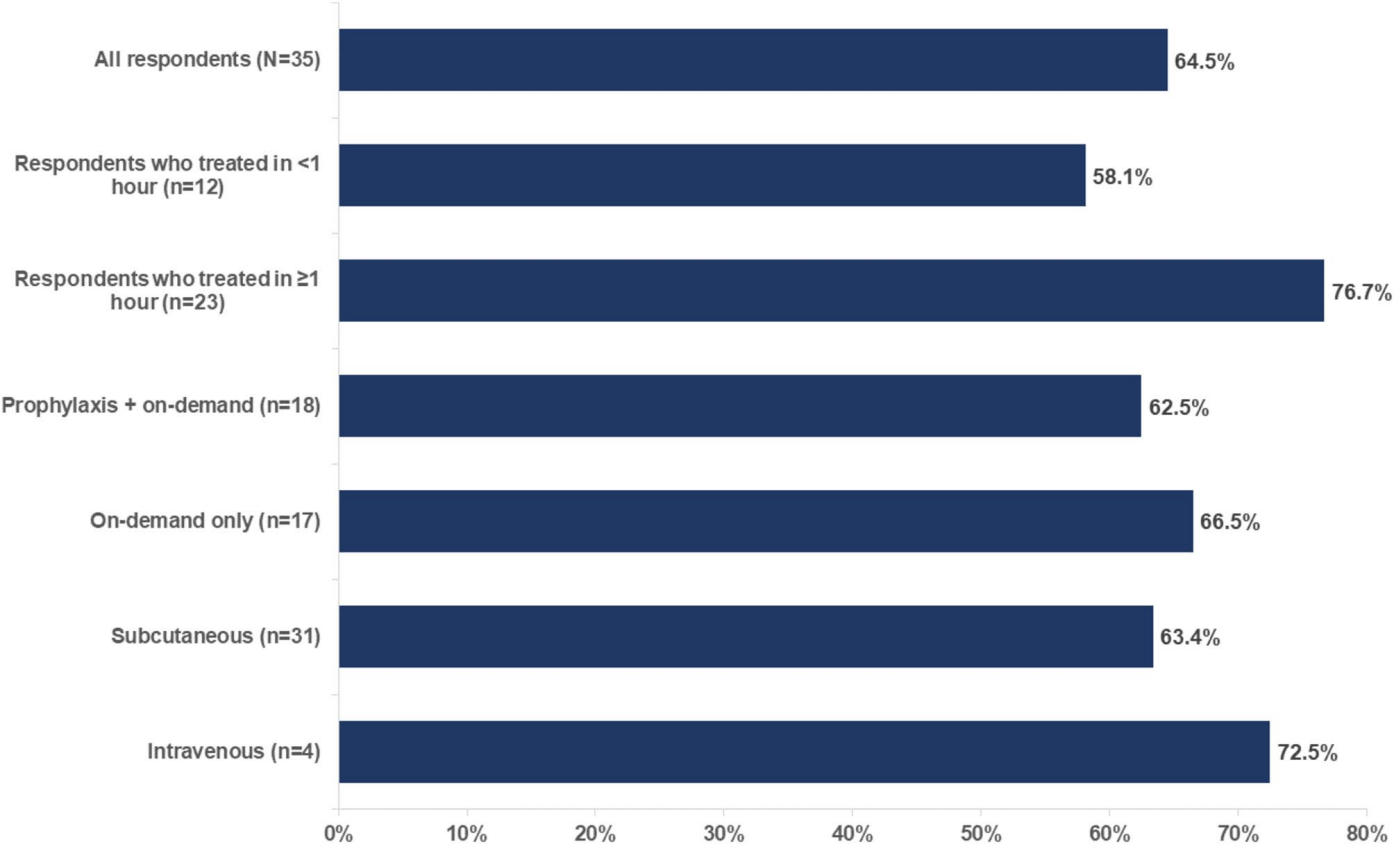
Percentage of respondents^a^ reporting that one additional dose of on-demand treatment was required to manage attack return ^a^All respondents were those who experienced return of HAE attack after taking on-demand treatment

When asked what percent of the time they took a second dose upon attack return after taking an initial dose of on-demand treatment, the mean percentage among all respondents who experienced return of HAE attack was 64.5% (Fig. 5). The mean percentage among respondents who waited less than 1 h was 58.1% versus 76.7% for those who waited 1 h or longer.

Of those who took more than one dose of on-demand treatment (*n* = 33), 87.9% required one additional dose to manage attack return. This percentage was similar for people using prophylaxis + on-demand therapy and those using on-demand therapy only (87.5% vs. 88.2%).

### Effects of early treatment compared with delayed treatment or non-treatment of HAE attacks

As previously reported, 75% of respondents reported that their HAE attacks were more severe when on-demand treatment was delayed [12] (82.6% of respondents who waited less than 1 h before initiating on-demand treatment reported that their attacks were more severe when delaying treatment versus 68.9% of those who waited 1 h or longer). Based on previously published results, 80% of respondents agreed that it took longer to recover from an HAE attack when on-demand treatment was delayed [12] (89.1% of respondents who waited less than 1 h before initiating on-demand treatment reported that it took longer to recover from an HAE attack when delaying treatment versus 73.8% of those who waited 1 h or longer).

**Fig. 6:**
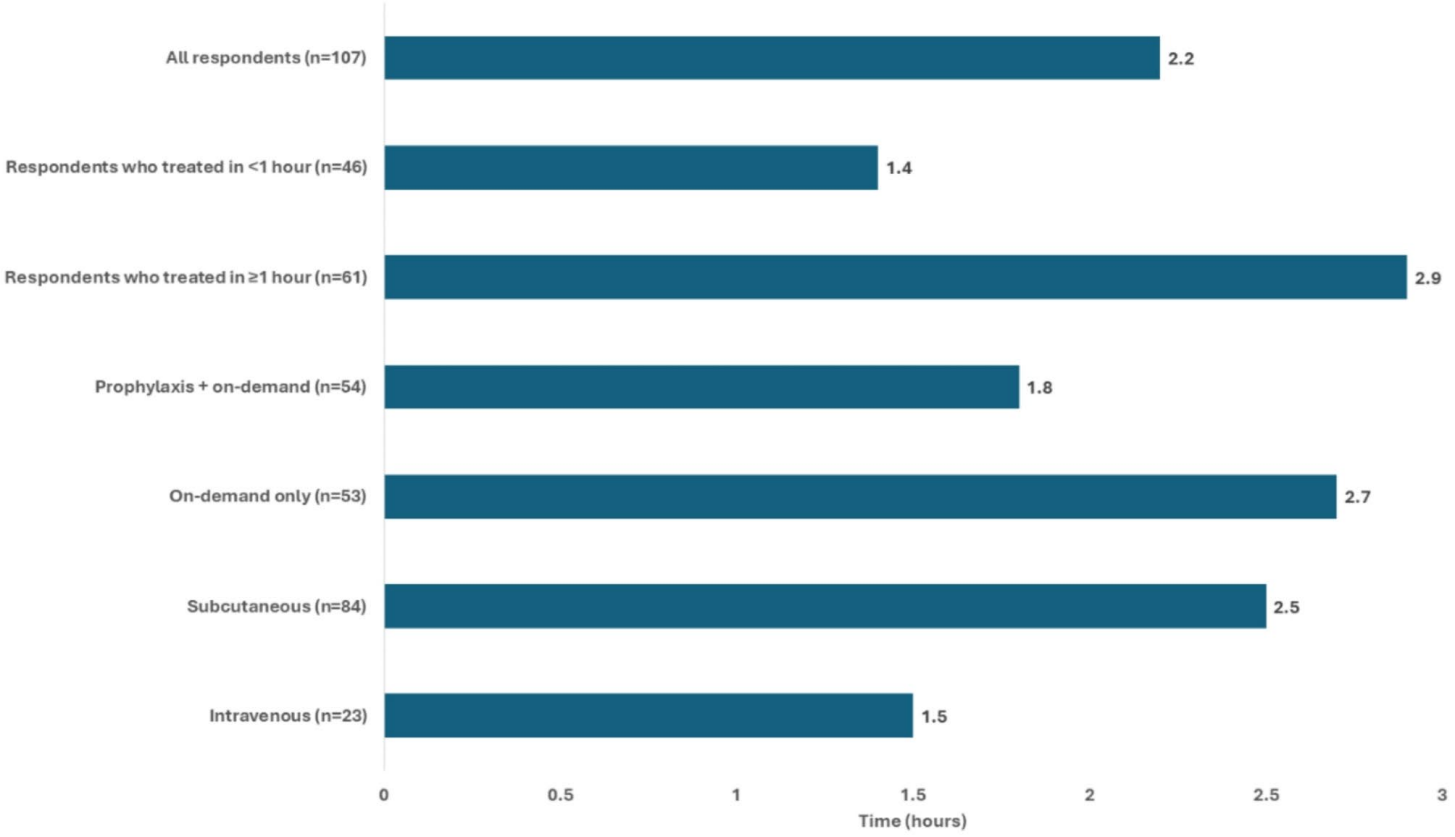
Mean time for respondents to feel in control of their HAE attacks after on-demand treatment

For those on icatibant the average time to treat was 2.65 h, and for those on C1-INH the average time to treat was 1.29 h. When asked how long it usually takes to feel in control of their HAE attacks after on-demand treatment, respondents reported a mean time of 2.2 h (SD: 5.1) post-treatment, with those who treat early (less than 1 h) reporting 1.4 h (SD: 1.1) and those who delay on-demand treatment reporting 2.9 h (SD: 6.7) (Fig. 6).

In clinical trials, the median time to symptom relief was 2.5 h with icatibant and up to 1.2 h with C1-INH [13, 14].

**Fig. 7:**
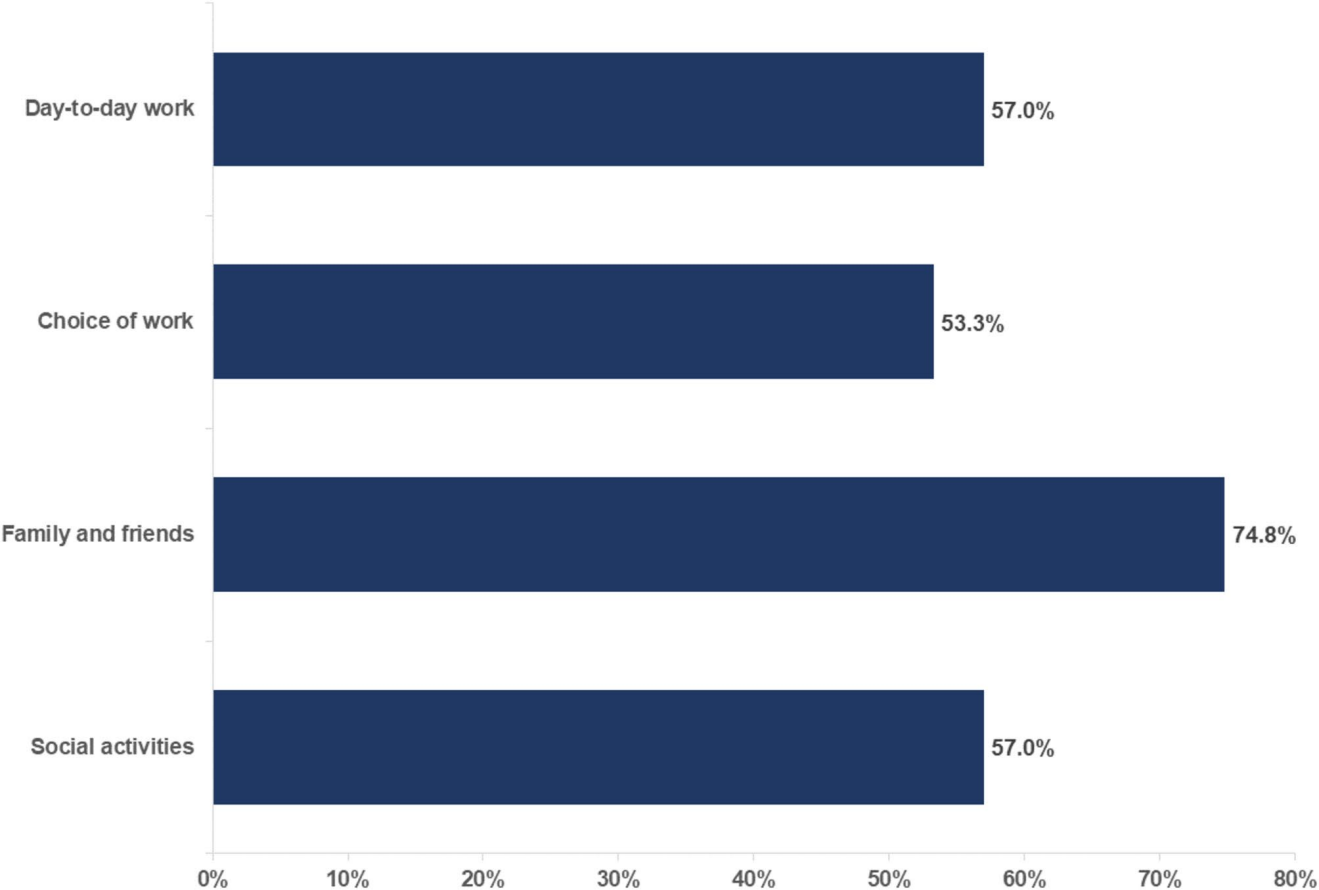
Percentage of respondents reporting that their on-demand therapy impacted their work participation, family and friends, and social activities

Respondents were asked if their on-demand therapy impacted their work participation, family and friends, and social activities (Fig. 7).

Among those who reported an impact on day-to-day work, 54.3% reported treating within 1 h of an attack and 59.0% reported waiting 1 h or longer. Those who reported an impact on choice of work were more likely to treat within 1 h of an attack (58.7%) compared with those who waited 1 h or longer (49.2%). A similar trend was seen in respondents who reported an impact on family and friends (76.1% reported treating within 1 h of an attack; 73.8% reported waiting 1 h or longer) and those who reported an impact on social activities (54.3% reported treating within 1 h of an attack; 47.5% reported waiting 1 h or longer).

## Discussion/Conclusions

The survey results highlight several key factors contributing to poor compliance with guidelines for on-demand treatment among individuals with HAE. Respondents who delayed treatment were more likely to express moderate or extreme anxiety about using on-demand treatment compared with those who treated immediately (59% vs. 34.7%). Among the reasons cited by respondents for delaying treatment were the attack not being considered severe enough to treat (86.9%), not having on-demand treatment with them (59%), and ​on-demand treatment being too painful (32.8%). Respondents who waited 1 h or longer to initiate on-demand treatment were also less likely to carry on-demand treatment when away from home and more likely to not treat their attacks compared with patients who treated within [< ] 60 min of an attack. Reasons given for not carrying treatment were the preference to treat at home (72%), forgetfulness (56%), excessive confidence in the effectiveness of long-term prophylaxis (77% of patients taking long-term prophylaxis), and avoidance of triggers (44%). These findings highlight the lack of compliance with HAE guidelines and the importance of reinforcing the need to carry on-demand treatment at all times, regardless of whether or not additional long-term prophylaxis is used [3].

Although HAE guidelines recommend that attacks be treated as early as possible [3], results from the current analysis confirm that patients frequently delayed on-demand treatment, which suggests that they may not fully understand the time window associated with the recommendation of “early treatment” or that barriers to treatment associated with current therapies are high for many patients. Many reasons for non-compliance with HAE guidelines may be associated with aspects of current on-demand treatment. Previous survey results indicated that respondents waited to treat HAE attacks due to various treatment-related reasons including pain associated with on-demand treatment, lack of privacy, time required to prepare on-demand treatment, and fear of needles [11]. These treatment-related factors may contribute to patient anxiety and logistical barriers associated with delaying on-demand treatment. Having treatment options that are more easily transportable, accessible, and less invasive can potentially remove many of these barriers to timely administration of on-demand treatment.

HAE is unpredictable, and any attack may be followed by another one in short succession; therefore, it is essential that patients have on-demand medication to treat all attacks and that all patients have and carry on-demand medication for the treatment of at least two attacks [3]. It is not uncommon for people with HAE to experience the return of an HAE attack requiring one or more additional doses of on-demand treatment, as previously reported by one-third of survey respondents [10]. Furthermore, survey results showed that initial delays in HAE attack treatment resulted in increased frequency of attack return when patients waited 1 h or longer to initiate on-demand treatment.

The HAE attack journey is highly individualized for each patient, marked by significant variability in the severity and frequency of attacks which may also fluctuate over a patient’s lifetime [11, 15]. As reported previously, respondents recognized the importance of recovering from an HAE attack quickly and agreed that their HAE attacks were more severe and longer when they delayed using on-demand treatment [12]. However, various factors can impede a patient’s ability to comply with HAE guidelines. Although HAE guidelines advise treating all attacks regardless of severity, patients may have misconceptions regarding attack severity, leading them to delay treatment when attacks are deemed not severe enough to treat. Although progression patterns may be variable, HAE attacks typically start as mild, then may progress in severity as well as move to other locations in the body [16].

Conflicting language contained in HAE guidelines may further contribute to the delayed treatment of HAE attacks. The recommendation from HAE guidelines to consider all attacks for on-demand treatment potentially conflicts with their guidance to treat attacks as early as possible. The term “consider” suggests the possibility of treatment delay as patients weigh the severity of the attack to determine whether or not to take action. Therefore, recommendations to enhance patient-physician discussions related to HAE guidelines should include engaging in a dialogue to more clearly define “early treatment” and encourage patient behavior that reinforces the importance of compliance with guidelines to improve patient outcomes. Utilizing patient-driven approaches to enhance communication between patients and physicians and facilitate shared decision-making can optimize outcomes by improving symptom relief, patient satisfaction, and adherence to treatment [17–19]. By asking appropriate questions, physicians can gather information about the patient’s goals, motivations, and other key factors to guide treatment selection [19].

One limitation of the analysis is that patient surveys rely on subjective opinions; nevertheless, they can offer valuable insights into the patients’ experiences and identify areas for improvement in care. The current analysis may also have limitations due to the potential for sampling bias in the online survey which was conducted in English and may not fully reflect the perspectives of a wider population, as it was conducted only in the US and respondents were predominantly female. Another limitation of the analysis is the inability to verify self-reported data. Furthermore, observational research is constrained by the lack of a control group for comparison.

In conclusion, survey results revealed that despite guidelines recommending early treatment many respondents do not treat HAE attacks immediately. Treatment delays were associated with increased attack recurrence and reduced likelihood of carrying on-demand medication. These survey findings support a tailored approach for the management of HAE to improve compliance with guidelines, encompassing open patient-physician communication that can help patients navigate the complex and highly individualized HAE attack journey and manage their attacks more effectively.

## Electronic supplementary material

Below is the link to the electronic supplementary material.

Supplementary Material 1

## Data Availability

Data is provided within the supplementary information files.

## Abbreviations

C1-INH: C1 esterase inhibitor
HAE: Hereditary angioedema
