# Supplementary material for: Factors contributing to non-compliance with on-demand treatment guidelines in hereditary angioedema
Source: Allergy Asthma Clin Immunol. 2025 May 21;21:25. doi: 10.1186/s13223-025-00969-0 (PMC12093744; doi:10.1186/s13223-025-00969-0)
Supplement: Supplementary file 1 — Supplementary Material 1 [file 13223_2025_969_MOESM1_ESM.docx]

**HAE Patient Survey**

**Survey Domains**

1. Attack Action Plan

2. Recognizing the First Signs of an Attack

3. Deciding Whether to Treat

4. Use of On-Demand Treatment

5. Impact on Activities of Daily Living and Anxiety

6. Potential On-Demand in the Future

**Background**

1. What is your age? (Specify)
   1. ____ years
2. What is your gender? (Select one)
   1. Male
   2. Female
3. Which of the following **prophylactic treatments** are you currently taking? (Select one)
   1. Cinryze
   2. Haegarda
   3. Takhzyro
   4. Orladeyo
   5. Androgens/steroids
   6. I do not take prophylactic treatment
4. What is your primary **on-demand treatment** you currently take? (Please select your primary on-demand treatment)
   1. Firazyr/Icatibant
   2. Ruconest
   3. Berinert
   4. Kalbitor

5a. What other on-demand treatments do you currently take, if any? (Select all that apply)

1. Firazyr/Icatibant
2. Ruconest
3. Berinert
4. Kalbitor
5. None of the above

**Section 1: Attack Action Plan**

1. What percent of the time do you carry an HAE on-demand treatment with you when you are away from your home? (Specify)
   1. ____ % of time
2. Approximately how far (time) will you travel from home without taking on-demand treatment with you? (Specify)
   1. _____ hours _____ minutes
3. What are the most common reasons you do not take on-demand treatment with you when away from home? (Please select up to 5 reasons in order of importance).
   1. It is too bulky
   2. I forget to take it with me
   3. It is embarrassing to carry
   4. I don’t want to have to check a bag at the door (cloak/coat room)
   5. I don’t want to have my bag checked by security (e.g., at an airport or concert)
   6. I avoid my triggers
   7. I would rather treat at home
   8. I am afraid I might leave it somewhere
   9. I take my prophylactic treatment, so I am confident I won’t have an attack
   10. Other (please describe)______
   11. Other (please describe)______
   12. Other (please describe)______

How many **minutes** does it take for you to prepare and administer a complete dose of your on-demand treatment? (Specify)

____ minutes [Range: 1 – 100]

9a. What percent of your HAE attacks do you **NOT** treat? (Specify)

____ % of attacks **NOT** treated [Range: 0 – 100]

1. If you do not treat an HAE attack, typically what percentage of the time will your plans for the day change? We understand this will vary depending on how severe the attack is, so please make your best estimate. (Specify)
   1. ___ % of time

**Section 2: Recognizing the First Signs of an Attack**

1. Many patients are able to anticipate when they are going to have an HAE attack. How clearly does the phrase “signs and symptoms” describe the first feelings that come before an attack? (Select one)

| Not at all well | Slightly well | Well | Very Well | Extremely Well |
| --- | --- | --- | --- | --- |
| 1 | 2 | 3 | 4 | 5 |

1. What other words or phrases do you use to describe the first feelings that come before an attack? [Unaided]
   1. ______
   2. ______
   3. ______
   4. ______
   5. ______

**Section 3: Deciding Whether to Treat**

1. When you experience the first signs and symptoms of an attack, on average how long do you wait before you initiate on-demand treatment? (Specify)
   1. _____ hours _____ minutes
2. Please imagine you are experiencing the first signs and symptoms of an attack. What are the reasons you **would initiate** on-demand treatment? (Unaided)
3. _____________________
4. Please imagine you are experiencing the first signs and symptoms of an attack. What are the reasons you **would not** initiate on-demand treatment? (Unaided)
   1. _____________________
5. Is access to a private area for injecting on-demand treatment a barrier to treating attacks quickly? (Select one)
6. Yes
7. No
8. Please think about a typical HAE attack you **do not immediately treat**. What are the most important reasons you might wait to treat an HAE attack? Please select up to 5 reasons in order of importance.
9. The attack is not severe enough to treat
10. I do not have on-demand treatment with me
11. I do not have any important activities that will be disrupted by the attack
12. I do not have a suitable/private area to administer my on-demand treatment
13. I have a fear of needles
14. My on-demand treatment is too painful (injection or burning)
15. I do not have the necessary time required to prepare for on-demand treatment
16. Important activities will be disrupted by treatment
17. Anxiety associated with refilling my on-demand treatment quickly
18. Cost of on-demand treatment
19. Other (please describe) ______________
20. I always immediately treat my HAE attacks [Exclusive]
21. Now, please think about an HAE attack that you **never treat**. What are the most important reasons you choose not to treat? Please select up to 5 reasons in order of importance.
    1. The attack is not severe enough to treat
    2. I do not have on-demand treatment with me
    3. I do not have any important activities that will be disrupted by the attack
    4. I do not have a suitable/private area to administer my on-demand treatment
    5. I have a fear of needles
    6. My on-demand treatment is too painful (injection or burning)
    7. I do not have the necessary time required to prepare for on-demand treatment
    8. Important activities will be disrupted by treatment
    9. Anxiety associated with refilling my on-demand treatment quickly
    10. The cost of on-demand treatment
    11. Other (please describe) ______________
    12. I always treat my HAE attacks

**Section 4: Use of On-Demand Treatment**

1. Please rate your level of anxiety when you anticipate using your current on-demand treatment. (Select one)

| Not Anxious |  |  |  |  |  |  |  |  |  | Extremely Anxious |
| --- | --- | --- | --- | --- | --- | --- | --- | --- | --- | --- |
| 0 | 1 | 2 | 3 | 4 | 5 | 6 | 7 | 8 | 9 | 10 |

1. What is making you feel anxious? Please select up to 3 reasons in order of importance.
   1. The injection needle
   2. Firazyr/Icatibant is painful
   3. I am unsure if the treatment will work quickly
   4. I am unsure if the treatment will work effectively
   5. I am unsure if there will be a rebound effect (my attack will return later)
   6. I do not have access to a suitable/private area
   7. I do not want to disrupt a current or planned activity
   8. Other (please describe ______________)

**For the following questions please respond based on the current on-demand treatment you are currently taking.**

1. Have you experienced attacks coming back or returning after taking your on-demand treatment? (Select one)
   1. Yes
   2. No
2. What percent of the time do you experience a return of your HAE attack after taking your on-demand treatment? (Specify)
   1. ____ % of time [Range starts 1%]
3. When an attack returns after taking your on-demand treatment, what percent of the time do you take a second dose of an on-demand treatment? (Specify)
   1. ____ % of time
4. When you take a second dose of an on-demand treatment, what percent of the time do you take a **different** on-demand treatment as the second dose? (Specify)
   1. ____ % of time
5. When you treat an attack more than once, on average, how many **more times** do you have to treat for it to resolve? (Select one)
   1. 1 more time
   2. 2 more times
   3. 3 more times
   4. More than 3 times
6. Are there certain attack locations that are more likely than others to return after taking your on-demand treatment? (Select one)
   1. Yes
   2. No
7. What attack locations are most likely to return? (Select all that apply)
   1. Face
   2. Airway
   3. Peripheral (e.g., hands, feet, etc.)
   4. Abdominal/stomach
   5. Genitals
   6. Other (please describe) _______
8. Are you able to recognize when you are starting to recover from an HAE attack? (Select one)
9. Yes
10. No
11. What are your cues that you are starting to recover from an HAE attack? (Select all that apply)
    1. The pain begins to subside
    2. The swelling starts to subside
    3. I start to feel a little better
    4. I feel less anxious
    5. Other (please describe) ________
12. How do you know you are **in control** of your attack after on-demand treatment? (Unaided)
    1. _______________________
13. How long (in hours) does it usually take to feel **in control** of your attack after on-demand treatment? (Specify)

Note: if it is less than one hour, please use a decimal (e.g., 0.5)

- 1. ______ hours [Range: 0.5 – 999]

1. How long (in days) does it take for you to **fully recover** from an HAE attack after on-demand treatment? (Specify)

Note: if it is less than one day, please use a decimal (e.g., 0.5)

- 1. ______ days [Range: 0.5 – 999]

1. Do you agree with the following statements? (Select one per row)

|  | Yes | No |
| --- | --- | --- |
| 1. My HAE attacks are more severe when I delay using my on-demand treatment | o | o |
| 1. It takes me longer to recover from an HAE attack when I delay using my on-demand treatment | o | o |
| 1. My level of anxiety decreases once I realize I am recovering from my HAE attack | o | o |

**Section 5: Impact on Activities of Daily Living**

1. Because of HAE, what percent of the time do you feel less than 100% of yourself? (Specify)
2. ___ % of time
3. Is it important to you to recover quickly from an HAE attack? (Select one)
   1. Yes
   2. No
4. Why is it important? ([Unaided]
   1. ____
5. Do you agree with the following statements? (Select one per row)

|  | Yes | No |
| --- | --- | --- |
| 1. If I recover quickly from my HAE attack, I can usually participate in my planned activities for the day. | o | o |
| 1. My decision to treat or not treat my HAE attack impacts my family and friends, not just me. | o | o |

1. Does your on-demand HAE treatment impact each of the following? (Select one per row)

|  | Yes | No |
| --- | --- | --- |
| 1. School | o | o |
| 1. Work (day-to-day work) | o | o |
| 1. Work (choice of work) | o | o |
| 1. Sports (participation) | o | o |
| 1. Travel (destination) | o | o |
| 1. Social activities (participation) | o | o |

**Section 6: Potential On-Demand Treatment in the Future**

For the following questions, please assume that the FDA has approved an **on-demand pill/tablet** that is as effective and fast-acting as your current preferred on-demand treatment.

1. What are the most motivating reasons for you to try taking a new on-demand pill/tablet for your HAE? (Select up to 5 reasons in order of importance)
   1. I prefer to take a pill/tablet versus an injection
   2. I can easily carry a pill/tablet with me at all times
   3. I can take a pill/tablet anywhere and do not need a private space
   4. I can take a pill/tablet on my own without help from others
   5. I can take a pill/tablet without disrupting the flow of my day
   6. I can take a pill/tablet without drawing attention to myself
   7. I can take a pill/tablet more quickly when I first recognize the signs and symptoms of a HAE attacks
   8. If I need a second dose, I can take it more quickly with a pill/tablet
   9. Other (please describe) ________

O [Opt-out] I am not interested in trying a new on-demand pill/tablet

1. What percent of the time do you think you would carry an effective HAE on-demand pill/tablet with you when traveling outside your home? (Specify)

____%

1. What percent of attacks do you think you would treat with an HAE on-demand pill/tablet? (Specify)
2. ____%
3. Would you treat your attacks faster/earlier with an HAE on-demand pill/tablet versus your current on-demand treatment? (Select one)

Yes

No

1. Why would you be more likely to treat an attack faster/earlier with an on-demand pill/tablet versus your current on-demand treatment? [Unaided]
2. ____
3. How would you describe your level of anxiety when anticipating using an on-demand pill/tablet compared to your current treatment? (Select one)

| Significantly  Less | Slightly  Less | Same | Slightly  More | Significantly  More |
| --- | --- | --- | --- | --- |
| 1 | 2 | 3 | 4 | 5 |

1. Would carrying an HAE on-demand pill/tablet at all times reduce your overall anxiety? (Select one)

| Not at all | Slightly | Somewhat | Very | Significantly |
| --- | --- | --- | --- | --- |
| 1 | 2 | 3 | 4 | 5 |

1. Are there attacks you would not treat with an on-demand pill/tablet? (Select one)
2. Yes
   - 1. What type of attacks would you choose not to treat with an on-demand pill/tablet? (Unaided) ________________________
3. No
4. Ideally, how many different places would you like to store an on-demand pill/tablet? (Specify)
5. ___ places [Range starts at 1]
6. Where would you most likely store an on-demand pill/tablet? (Select all that apply)
   1. Car
   2. Work bag
   3. School backpack
   4. Handbag
   5. Sports bag
   6. Locker (school or gym)
   7. Home
   8. Place of work
   9. School nurse
   10. Other (please describe) _____________
7. What are the most compelling reasons for trying a new treatment/therapy? (Unaided)
8. ____
9. Do you consent to quotes from your answers to the survey questions being used anonymously?
   1. Yes
   2. No
